# Presentiments

**Published:** 1886-07

**Authors:** 


### PRESENTIMENTS.

**Hall's Journal, July, 1886, vs. Hall's Journal, July, 1885.**

The leading article of the July 1885, number of HALL'S JOURNAL OF HEALTH, is entitled "A Presentiment," which the writer defines to be "an impression on the mind, that something is going to happen," and branching out from this very original and lucid definition, the writer in, if possible, a still more original manner runs riot with his subject, waxing betimes exceedingly funny and then settling down to business by ascribing to "weak-minded people," a monopoly of presentiments. It is just this sort of egotistical nonsense to which the intelligent and fair minded readers of the JOURNAL, in time past objected. Had the writer of this article of a year ago, made the slightest investigation beyond opaque materiality, he would have been better qualified to treat of a subject which, for all recorded time, has engaged the attention of the ablest of thinkers, and formed the subject of earnest philosophical inquiry. "There is," remarks Cicero, "an ancient opinion, drawn even from the heroical times (that is, from the utmost bounds of time spoken of,) that there is among men a certain devination, which the Greeks call prophecy, (or inspiration) that is, a *presension* and knowledge of future things."

Volumes have been written upon this interesting subject, with very many well authenticated facts and circumstances of presentiment, in such minute detail as fairly to challenge unbelief. The works of Catherine Crow and Robert Dale Owen, bear abundant testimony of this remarkable truth, yet, HALL'S JOURNAL (O. S.) refers them all to weak mindedness. We solemnly pledge our readers that under the present management no like balderdash shall be admitted to these columns. Denial is not proof, nor is ridicule an acceptable substitute for reason and investigation, nor indeed is any mere statement barren of facts, entitled to any weight *pro or con* propositions concerning which wise men differ. We are in search of truth. Lead where it will, we shall not falter by the way. It is our purpose in the near future, to treat of this subject in a manner suited to the present aims of the JOURNAL.

